# Evaluating the Feasibility of the Participant‐Led Video Intervention to Train Support Workers From the Perspective of Disability Sector Professionals

**DOI:** 10.1111/hex.70432

**Published:** 2025-09-17

**Authors:** Megan Topping, Jacinta Douglas, Kate D'Cruz, Di Winkler

**Affiliations:** ^1^ La Trobe University Melbourne Australia; ^2^ Summer Foundation Melbourne Australia

**Keywords:** disability support, intervention evaluation, participant‐led videos, people with disability, support workers, training evaluation

## Abstract

**Background:**

Tailored disability support is fundamental to the lives of adults with disability and complex needs. However, support quality is inconsistent. An intervention was co‐designed to help people with disability direct their supports: participant‐led videos (PLVs). Following a successful pilot study, this study aimed to evaluate the feasibility of the use of PLVs by sector professionals working with people with disability in the community.

**Methods:**

Sixteen sector professionals attended a training workshop on PLVs. Following the workshop, five sector professionals were funded to collaborate with people with disability to produce PLVs. The training workshop was evaluated using an online survey (*n* = 15) and a 1‐month follow‐up interview (*n* = 8). Post‐production interviews (*n* = 4) were conducted to evaluate the experience of producing a PLV.

**Results:**

All sector professionals endorsed the training workshop and reported high levels of confidence in producing videos following the workshop. Highlights of the workshop included the centrality of the voice of people with disability and the design and quality of the content. All participants planned to implement what they had learnt and create videos with people they worked with. The experience evaluation findings were also positive, with all sector professionals endorsing the usefulness of PLVs as a tool to capture genuine behaviours and enable disability support workers to get to know the individual. Sector professionals saw the value of the intervention in helping to give people with disability a voice and minimise misrepresentation of individuals.

**Conclusions:**

This study demonstrates the feasibility of PLVs outside of the team that developed the intervention. This evaluation contributes to an evidence base for funding to facilitate the development of PLVs as a tool to help improve the quality of support and maximise autonomy for people with complex needs. Future research is required to evaluate the longer‐term impact of PLVs on support quality.

## Involvement of People With Lived Experience

People with disability and their families were integral to the co‐design and piloting of the Participant Led Video (PLV) intervention, providing valuable insights to ensure the intervention accurately reflected the real‐world needs and priorities of those receiving support. Feedback obtained through interviews with people with disability, families and support workers helped improve the intervention and shape the resources designed to support people with disability and sector professionals in using the intervention [1]. A key part of this evaluation was informed by interviews with sector professionals working with people with disability. These insights have directly influenced the refinement of the PLV resources and training materials.

## 1 Introduction

Disability support plays a fundamental role in the lives of many adults with disability. For support to be of quality, it needs to be tailored to the needs and preferences of each person. The National Disability Insurance Scheme (NDIS) was introduced in Australia in 2013 to provide individualised supports to empower people with permanent and significant disability to live the life they choose [2]. However, a decade on from the introduction of the NDIS, people with disability are receiving support of variable quality [3, 4]. Indeed, some support providers and workers continue to practise within outdated models that do not centre the choice and control of people with disability [3, 4, 5]. Coupled with complex cognitive and communication impairments experienced by many people with disability, these outdated, sometimes paternalistic, practices can be especially damaging to the quality of disability support [3, 5, 6, 7].

Research into disability support illustrates that the interaction between the person with disability and their support worker is fundamental to quality support provision [8, 9, 10, 11]. To optimise the interaction, people with disability and support workers need to work well together, with respect and good communication, to facilitate quality support [8, 12]. Moreover, people with disability want the opportunity to choose and lead their support, and in turn, disability support workers must learn from the person they are supporting and provide tailored support accordingly [6, 8, 13]. However, people with disability have voiced that leading support workers can be difficult and tiresome [13, 14]. For example, people with disability have expressed that they have not been trained as employers, that some disability support workers have poor attitudes and are not willing to listen, and that repeating instructions can be burdensome [6, 14]. Further, it is critical to acknowledge that some people with complex cognitive and communication impairments do not have the capacity to lead their support workers in the moment [15].

The support sector has long been characterised as undervalued and often lacking formal training requirements [16, 17, 18]. In Australia, the introduction of the NDIS led to a substantial increase in demand for support, prompting an influx of unskilled workers into the sector [16, 19]. Coupled with the psychosocial occupational risks experienced by support workers [20], these conditions contribute to high turnover rates. As a result, continuity of support is often not available to people with disability [19, 21], and many NDIS participants report receiving support from unfamiliar workers [6, 22]. Opportunities for professional support and development remain inconsistent, particularly since support workers are employed via several avenues (e.g., via a service provider, an online platform, directly for the person with disability, or in supported‐living arrangements), often with limited dedicated funding for training [6, 21, 23, 24].

Despite the concerns centred around unsuitable workers entering the support workforce, there are mixed views in reference to mandatory registration and formal qualification requirements [25]. Formal qualifications for support workers are available in Australia, and the NDIS Quality and Safeguards Commission have specified online training requirements to become an NDIS registered support worker [26]. However, some people with disability, disability advocates and researchers are concerned that mandatory registration restricts the autonomy of people with disability to choose their support workers [25]. Moreover, arguably, the current qualifications available for support workers in Australia, for example, do not effectively prepare workers to provide quality support [21], and there is evidence suggesting people with disability would prefer to train support workers themselves [27]. In recent years, more evidence‐based training resources have been developed to help support workers deliver best practice in line with the needs and preferences of people with disability [28, 29, 30, 31, 32]. Yet, most resources are designed as an instructional or passive training tool with too few co‐designed with people with disability. Furthermore, there is a lack of resources directed at building the capacity of people with disability to lead their support workers [1, 28].

This literature highlights a tension in disability support: people with disability want to lead and train their support workers to ensure their needs and preferences are respected [6, 8, 13, 27], but this can be challenging or tiring, particularly for those with complex communication and cognitive impairments [13, 14, 15]. Simultaneously, the support workforce faces challenges in training, consistency and continuity, which can undermine person‐centred practice [6, 16, 19, 21, 22, 23]. These intersecting issues point to the need for co‐designed approaches that both empower people with disability to lead their support workers and enhance support workers' readiness to provide person‐centred support [1, 28].

To address this gap, we co‐designed a novel intervention—participant‐led videos (PLVs)—with people with disability, support workers and close others (e.g., family of people with disability) to help people with disability direct and train their support workers [34]. Drawing on principles of participatory research [35, 36, 37, 38], a conceptual framework was established that informed both the initial aim of PLVs—to centre the voice and agency of people with disability—and the methodology used to develop them through co‐design. PLVs are a tool whereby people with disability, with support from sector professionals where needed, lead the production of a video in which they tell support workers how they would like to be supported. Initial pilot research demonstrated promising outcomes [1], supporting further investigation of PLVs in practice. The current study extends this research by evaluating the feasibility of PLVs as a tool to be used in practice outside of the original project team. The first objective was to evaluate sector professionals' experience of training on PLVs and their likelihood to apply the training and recommend it to others. The second objective was to investigate the experience of collaborating with a person with disability to make a PLV. Both the training and the subsequent PLV development experience were evaluated with funding support from the National Disability Services (NDS) Workforce Impact Collective.

## 2 Methods

### 2.1 Overview of the PLV Intervention

Critical to the PLV intervention is an emphasis on building the capacity of people with disability to lead their support workers by creating a video in which support preferences are identified and communicated. Developing a PLV involves six steps: (1) goal setting, (2) scripting, (3) storyboarding, (4) filming, (5) editing and (6) sharing. The sixth step was added in response to the pilot evaluation findings [1]. This final step emphasises that the person with disability retains full ownership and control over their PLV, including decisions about its storage and access, keeping privacy and consent central. The process is supported by free, publicly available resources [39]. These resources include a *how‐to* video introducing the PLV concept, a workbook and accompanying video plan to guide people with disability through each step of making their video, a supporter guide for those assisting in the process (e.g., family, therapists and coordinators), a document offering strategies to support thinking and communication, and several sample PLVs to help users visualise the final product.

**Figure 1:**
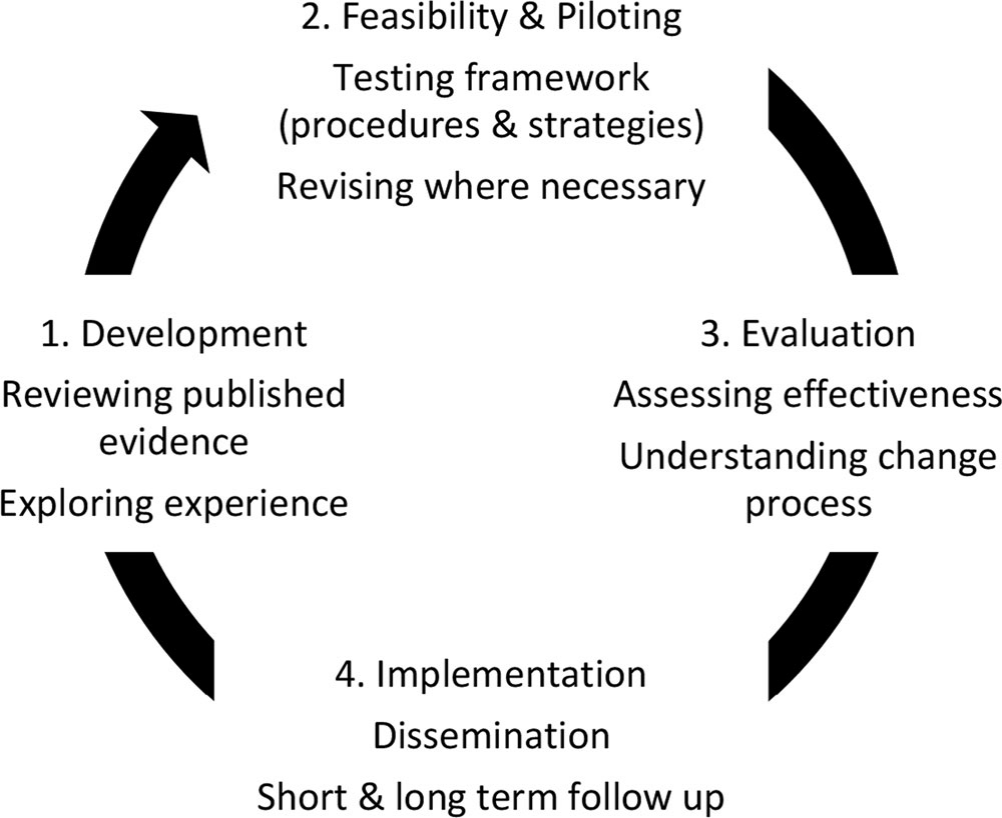
Process of development and evaluation: modelled after Craig et al. [40].

The project followed the Medical Research Council (MRC) guidance for developing and evaluating complex interventions. Specifically, the four‐phase approach (development, feasibility and piloting, evaluation, and implementation) for creating and assessing complex interventions is outlined by Craig et al. [40] (see Figure 1). The PLV intervention was initially informed by a scoping review of the literature, developed in partnership with people with disability, support workers and close others in co‐design workshops, and piloted with five individuals with acquired brain injury and cognitive‐communication impairments, along with their supporters and facilitators [1]. An independent mixed‐methods evaluation found high satisfaction across all participant groups and highlighted that PLVs provided a person‐centred, empowering experience that supported individuals to express their preferences, feel validated, and take greater control over their supports.

**Figure 2:**
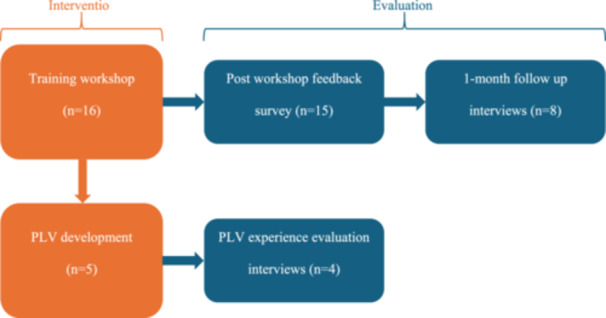
Process of intervention and evaluation.

This study built upon the pilot study [1] and explored the feasibility of PLVs to be used in practice by evaluating a PLV training workshop from the perspective of sector professionals and subsequently evaluating the experience of sector professionals producing a PLV [1, 8, 39] (see Figure 2 for an overview of the intervention and evaluation process). The training workshop was conducted by the Summer Foundation, and the research evaluations by La Trobe University. The evaluation outlined in this paper utilised a mixed‐methods approach to capture both the breadth of participant responses through quantitative ratings and the depth of experiences through qualitative feedback. This included a two‐stage evaluation of the training workshop (Stage 1: immediate survey and Stage 2: 1‐month follow‐up interview), allowing for both immediate and reflective, more in‐depth feedback. Additionally, a third stage of the evaluation explored sector professionals' experiences producing PLVs in practice, providing insight into the implementation experience beyond the training context. This approach enabled triangulation and a more comprehensive understanding of the feasibility of the PLV intervention [41]. The university ethics committee granted approval for the independent evaluations.

### 2.2 Training Workshop and Subsequent PLV Production

The NDS' Workforce Impact Collective funded the Summer Foundation to conduct a 1‐day training workshop with disability sector professionals in the Australian Capital Territory (ACT). Facilitators from the Summer Foundation, who were involved in the development of PLVs, hosted the training workshop. Disability and health sector professionals working with adults with any disability type were invited via the Summer Foundation's network of ACT service providers, hospitals, advocacy organisations, government contacts and non‐profit organisations. Invitations were sent via email and phone calls from an administrative staff member, as well as social media advertisements. The workshop was free to attend. During the workshop, sector professionals were trained in the six‐step PLV process and participated in a practical activity to create a trial PLV using their mobile phones. Sixteen sector professionals who worked with adults with a range of developmental and acquired disabilities attended the workshop (five support coordinators, three managers, two occupational therapists, two disability support workers, one social worker and three other roles) from nine different organisations, including advocacy services, hospitals, service providers and community support services.

At the conclusion of the workshop, the sector professionals were invited to submit a funding application to the Summer Foundation to support them in trialling the use of the PLV intervention in their practice. Five trained sector professionals (4 females and 1 male) submitted applications, and all were approved for funding. Each sector professional was funded $1500AUD (per video) for their time to collaborate with a person with disability with whom they worked to produce a PLV within a 4‐week time frame. Two of the professionals collaborated with two people, meaning seven videos were produced in total. The people with disability were all NDIS participants with cognitive and communication impairments and identified as having an intellectual disability (3), autism (2), acquired brain injury (1) or Down syndrome (1). Videos were completed using the PLV resources, independent of the training facilitators or researchers.

### 2.3 Training Workshop Evaluation

A mixed‐methods evaluation of the training workshop was conducted with the use of qualitative and quantitative measures. The evaluation had two components: Stage 1: an immediate post‐workshop survey and Stage 2: a 1‐month post‐workshop telephone interview. The aim was to evaluate the PLV training workshop from the perspective of sector professionals to inform further development of the training and resources. Participation in the survey and the interview was voluntary, with all workshop participants invited. The survey link was provided at the conclusion of the workshop, with an option to provide details to be contacted about an interview following the survey.

#### 2.3.1 Post‐Workshop Survey

The immediate post‐workshop evaluation survey was custom‐designed by the researchers and conducted via an online polling platform (https://www.slido.com). Slido complies with the General Data Protection Regulation (GDPR) and outlines its data protection and privacy policies on its website. All participant responses were anonymous, and no personally identifiable information was collected during the survey process. The survey items included customised 5‐point rating scales (responses ranging from 1 poor to 5 excellent) covering (i) understanding of PLVs pre‐ and post‐workshop; (ii) confidence to use the resources and (iii) likelihood to collaborate with someone with disability to create a PLV. Open‐ended questions explored the usefulness of the training, suggestions for improvement, actions following the workshop, and additional feedback. All survey respondents completed consent to participate via the survey link. Completion of consent opened a separate survey link so that survey responses remained anonymous. See Appendix 1 for the full survey.

#### 2.3.2 1‐Month Post‐Workshop Follow‐Up Interviews

A female researcher (M.T.) with experience interviewing adults with and without disability, independent from the workshop facilitators, conducted semi‐structured telephone interviews approximately 1 month after the training workshop. The researcher attended the workshop to present on the evaluation and met the participants, but had no prior relation with them. Before commencing the interview, the sector professionals provided informed written consent to participate.

The interview guide included the use of customised rating scales and open‐ended questions. Five‐point customised rating scales measured satisfaction, usefulness and enjoyment of the training workshop, confidence to support someone with cognitive and communication impairments to produce a PLV following the workshop and the overall quality of the workshop (responses ranging from 1 very low to 5 very high). Open‐ended questions were used to gain further insights into the workshop experience and future use of PLVs. See Appendix 2 for the interview schedule.

### 2.4 PLV Experience Evaluation

The experience of developing a PLV outside of the training workshop was evaluated using a mixed‐methods design (Stage 3). The aim was to evaluate the experience of collaborating with a person with disability to develop a PLV and the implementation of the training in practice. Participation in the evaluation was voluntary, and the five sector professionals were invited to participate.

Semi‐structured interviews were conducted by a researcher, M.T., independent from the training workshop facilitators, via telephone within 1 month of the production of the PLVs following provision of written informed consent. Sector professionals were asked to describe their experience of each step of the PLV development process and to rate their satisfaction with, and enjoyment of each step, as well as overall satisfaction with and usefulness of the PLV on customised rating scales with a 5‐point response format (1 very low to 5 very high). In addition, they were asked to rate how likely they would be to recommend the process to others on a 10‐point response format (1 very unlikely and 10 very likely). See Appendix 3 for the full interview schedule.

### 2.5 Data Analysis

Quantitative data from customised rating scales in the post‐workshop survey and the 1‐month post‐workshop follow‐up interviews were analysed descriptively using means and standard deviations to summarise participants' perceptions of the training and PLV process. The interview data collected from the 1‐month post‐workshop and the experience evaluation interviews were analysed in line with Braun and Clarke's six‐step thematic analysis approach using NVivo software [42, 43]. Interviews were audio recorded and transcribed verbatim, and transcripts were de‐identified before analysis by the researcher. Following familiarisation with the transcripts, the first author (M.T.) conducted initial coding inductively with codes generated directly from the data. Regular analytical discussions took place with the second author (J.D.). Using a process of iterative comparison, the first and second authors identified themes by looking for broader patterns across the codes, with the use of memo‐writing to support theme development. Quantitative and qualitative data were integrated during analysis by comparing and interpreting findings across both datasets to identify areas of convergence and divergence.

## 3 Results

### 3.1 Training Workshop Evaluation

Fifteen of the 16 sector professionals who attended the training workshop completed the anonymous evaluation survey immediately post‐workshop via the online platform, Slido. Their roles included: support coordinators (4), managers (3), occupational therapists (2), disability support workers (2), social workers (1) and other roles (3). Providing occupation information was optional, and no further demographic information was collected for the survey participants to reduce the risk of social desirability biases if participants felt identifiable. Eight of the 15 trained sector professionals (female = 5 and male = 3) consented to participate in 1‐month follow‐up telephone interviews. The interviews ranged from 15 to 45 min.

#### 3.1.1 Quantitative Findings

##### 3.1.1.1 Post‐Workshop Feedback Survey

Participants retrospectively reported their pre‐workshop understanding as fair‐to‐good (mean 2.73) compared to their post‐workshop understanding as very good‐to‐excellent (mean 4.27).

Mean confidence ratings after the workshop were good‐to‐very good (confidence to use resources 3.87 and confidence to support someone to create a PLV 3.73). Ten participants expressed that it was likely they would collaborate with someone to create a PLV in the next 3 months, and the remaining five participants said it was possible. All participants said they were likely to (*n* = 5) or would definitely (*n* = 10) recommend the training to other sector professionals.

##### 3.1.1.2 1‐Month Post‐Workshop Follow‐Up Interviews

All eight interview participants intended to collaborate with people with disability to produce PLVs within the next year, and five had already started the process. Average ratings for satisfaction, usefulness, enjoyment, quality of the workshop, and confidence to support someone to make PLVs were consistently positive across the eight participants (≥ 4.0) (see Table 1).

**Table 1:** Post‐workshop follow‐up interview ratings.

Rating	Mean	Std. deviation
Satisfaction with the workshop	4.50	0.756
Usefulness of the workshop	4.50	0.756
Enjoyment of the workshop	4.75	0.463
Confidence to support a participant to make a PLV	4.00	0.535
Overall quality of the workshop	4.38	0.744

#### 3.1.2 Qualitative Findings

This section combines the qualitative results from the post‐workshop survey open‐ended questions and the 1‐month post‐workshop follow‐up interviews. The analysis produced five main themes, which are explored below. Supporting quotes are provided in Table 2.

**Table 2:** Training workshop evaluation—participant quotes (interview and survey data).

Theme	Quotes
Theme 1: Workshop highlights	‘The training was so well designed and felt really professional as well. The resources are incredible. They're really well laid out and easy to interpret for both, for the consumer and for the service, so I think it's a really great job…. Also I found exploring somebody's story really powerful and another way of looking at how you can determine values and goals’. PPT04 ‘…Getting the chance to put what we were learning into action straight away…. I felt really valued by the high quality of the presenters, so Summer had obviously put some of its best people in that room with us and it just made me feel like it was really important training, like that it was really valued … that kind of motivated me to also invest my resources into it’. PPT06
Theme 2: Workshop improvement suggestions	‘I don't know if it was possible to have a participant there that had made a video or something. That may have been good’. PPT02 ‘There wasn't clarity between which workbooks you were working from. So, whether it be from the participant, whether it be from the project manager, the coordinator side of things. And there weren't references in between, so it was quite jumping backwards and forwards. I think that it was just getting familiar with the documents’. PPT07
Theme 3: Post‐training actions	‘I'm definitely going to make some videos. I'm going to try and figure out how to carve out some time to do the scripting, filming and editing…’. *Anonymous survey response* ‘I am going to change my language and the way I gather information…. I am going to ask my clients if the PLV is something they are interested in completing’. *Anonymous survey response*
Theme 4: Barriers to implementation	‘…Time. Resources. So, a lot of our people are one‐on‐one supported so you'd need one person to work with a person plus a person to film. Yeah, and communication barriers with a lot of them’. PPT02 ‘I think communication challenges would also be a barrier if you didn't know the client well or you couldn't communicate with them effectively’. PPT03
Theme 5: How PLVs will help clients	‘…It's really genuine coming from them to the new person in their life to understand how to better support the person because it's coming from them so I think that's really powerful’. PPT01 ‘I think it would improve the accountability of the different staff members that they have coming into their home and I also think that it allows them to communicate with staff without actually having to verbally communicate in the present, which is quite tricky for a lot of my clients’. PPT03

##### 3.1.2.1 Theme 1: Workshop Highlights

Sector professionals remarked on the quality of the design and professionalism of the training workshop and specifically appreciated how practical it was. Professionals commended the quality of the facilitation and commented that they liked having the variety of three workshop facilitators. Some sector professionals considered the training of specific steps in the PLV process the most useful part of the workshop, including the scripting and storyboarding stages, goal setting and editing. Other professionals commented on the use of storytelling as a workshop highlight and valued having the genuine voice of people with disability at the forefront of the training and resources. Additionally, the opportunity to connect with other people working in the sector with a shared interest in collaborating with people with disability to produce PLVs was appreciated. Finally, sector professionals endorsed the usefulness of the PLV training resources.

##### 3.1.2.2 Theme 2: Workshop Improvement Suggestions

The most common suggestions to improve the training workshop concentrated on technology and editing PLVs. Multiple sector professionals referenced the focus on Apple technology and suggested providing guides for other devices and software (e.g., Android). Another suggestion was to invite a person with disability who had produced a PLV to the workshop to share their experience of working with a sector professional in developing their PLV. Some professionals recommended providing clearer instructions in the workshop regarding which workbook was being referred to, and they suggested this could be improved by better cross‐references between the supporter guide and the workbook designed for people with disability [39]. Finally, a filming tip sheet resource was recommended with reminders, for example, to leave time lags between prompting and the person speaking.

##### 3.1.2.3 Theme 3: Post‐Training Actions

As reflected in the quantitative results, all sector professionals planned to implement the training and collaborate with people with disability with whom they work to support them to create videos. Some professionals also planned to practice editing, share what they had learnt with colleagues and complete the video started in the training workshop. Interestingly, one sector professional remarked that, in response to the training, they committed to adapting their use of language when gathering information with clients.

##### 3.1.2.4 Theme 4: Barriers to Implementation

The most frequently reported barrier was a lack of time or resources. Correspondingly, sector professionals reported that they would struggle to decide which clients to prioritise to collaborate with to develop a PLV. Another barrier anticipated by multiple sector professionals was obtaining consent from people with disability and their family members to collaborate on a PLV. Other identified concerns included editing skills and the use of software. Some professionals were concerned that working with people with significant communication impairments may make it more difficult to produce a PLV.

##### 3.1.2.5 Theme 5: How PLVs Will Help Clients

The sector professionals endorsed PLVs as a tool to give people with disability a voice in the delivery of their support and to communicate more effectively with their support staff. Sector professionals remarked that having a PLV would improve the accountability of support staff and could help understanding around dignity of risk issues. Correspondingly, multiple professionals referred to the videos as a storytelling tool that could help minimise misrepresentation of the individual, reduce the burden of people having to retell their story and help to reduce conflict between the person and their support worker.

### 3.2 PLV Experience Evaluation

#### 3.2.1 Participants

Four of the five trained sector professionals consented to participate in the evaluation. The four professionals were female and ranged in age from 29 to 49 years (mean = 41 years). They all worked in the ACT and included two occupational therapists, one chief executive of a disability advocacy organisation, and one service provider staff member. Interviews ranged from 25 to 45 min.

#### 3.2.2 Qualitative and Quantitative Findings

Thematic analysis of the interview data produced four key themes that captured the experience of making a PLV from the perspective of sector professionals who had undergone PLV training. Supporting quotes are provided in Table 3. The results of the customised scales to capture satisfaction and enjoyment are integrated in the discussion of the thematic findings.

**Table 3:** PLV experience evaluation—participant quotes (interview data).

Theme	Quote
Theme 1: Satisfaction and enjoyment	‘And looking at photos with him and making sort of suggestions and, you know, and I did a lot of connecting with him personally’. PPT07 ‘I just didn't have the time to do [editing], and I didn't think I had the skill, particularly for [client], because it was a lot of fine‐tuning because we had to cut out his mum's sections, but they were really tight, and we didn't leave enough space’. PPT01
Theme 2: Strengths of the PLV process	‘[Step 1] gave really great focus to our conversation because I certainly was talking to people that—I talk to them about all sorts of things, so it was really good to help us stay on track gathering the particular kind of information that we were going to need in the video. And it also opened up conversations that we've never had before, so I found that really helpful’. PPT06 ‘We did capture some really good footage of [person with disability] when, say, she was coming to her mum to ask for something and then capturing that interaction, and that was really, really good to have that, because then the support workers could see how well her mum interacts with her and vice versa, so then it's more likely to be modelled and taken up’. PPT01 ‘He just spontaneously burst into laughter and I'm like oh he likes it, and then we end up watching it together twice because it was just magic…. He was really, really, really happy with it and just kept saying, “Thank you so much”’. PPT07
Theme 3: Challenges experienced during PLV development	‘I found [goal‐setting] the most challenging because he has short‐term memory loss and he has a tendency to talk about one subject for a very long time … you don't want to interrupt someone because they have a feeling of purpose from being heard, but also then to try and redirect to the purpose of your conversation, so it can be extremely challenging … it is hard to get him to remember what we're there for and to keep redirecting backwards and then reminding him to write things down’. PPT07 ‘Script writing I think probably we could have done better. We were trying to think of “how do we make sure she can tell us all of this?” and making sure it's coming from her perspective. So that was tricky to do, and I don't know how well we did that, but I think this is like a learning process for us, with someone with really complex communication difficulties’. PPT01
Theme 4: What participants plan to use PLVs for	‘Sometimes her communication is hard to understand and her different behaviours, they really wanted to help educate sort of or train the support staff to feel more comfortable and more familiar with how to best support her’. PPT01 ‘They were really keen to watch it because they had a job interview panel coming up where they had a bunch of people applying for a job as a support worker, so they were like, oh, this fits in really well with what we're doing for [client]’. PPT06 ‘I think it's a good start and I'm hoping that it will prolong her stay at this day program ‘cause she's been through quite a few just because of how complex she is, so—or misunderstood is probably a better word’. PPT03

##### 3.2.2.1 Theme 1: Satisfaction and Enjoyment

Mean satisfaction and enjoyment ratings with the goal setting, scripting and film steps were high‐to‐very high (> 4). The average enjoyment rating for the storyboarding step was high (4.1), but the average satisfaction rating was slightly lower (3.8). Qualitative findings suggest sector professionals were slightly less satisfied with the storyboarding step due to time constraints. However, storyboarding was still highly enjoyable because it enabled the sector professionals to connect with clients and their families. The inverse was true for editing, with a high mean satisfaction rating (4.4) but a lower mean enjoyment rating (3.6). Comments made by the sector professionals suggested that, due to their unfamiliarity with editing, they found it challenging and time‐consuming. Despite the difficulties, however, the sector professionals were pleased with the video produced, hence the high satisfaction ratings. The final step, sharing the video, was not rated by the sector professionals as they had not all reached this point before the interview. Overall mean satisfaction and usefulness ratings of the finished video were very positive (satisfaction 4.4; usefulness 4.8). All participants expressed that they would be very likely (rating 10 on a 10‐point scale) to recommend PLVs to others.

##### 3.2.2.2 Theme 2: Strengths of the PLV Process

The sector professionals found the steps easy to follow and commented on the usefulness of the training workshop and resources in guiding the process. Additionally, the professionals remarked on how fun the process was, mostly highlighting the goal‐setting and filming steps. Considering each step individually, sector professionals commented that the first step, goal setting, was of particular importance to the success of the PLV as it gave focus to the conversation, complemented by the second step, scripting, to fit the pieces together ‘like a puzzle’. Family and support worker input was highly valued in the storyboarding and filming steps, especially for providing content such as photographs and videos, as well as assisting with filming activities in the home. Considering the importance of portraying the person authentically, sector professionals found it helpful to edit the video themselves and send drafts to the person with disability and their family to review and provide feedback. Sector professionals reported that people with disability enjoyed the filming and were proud of the video. Overwhelmingly, the sector professionals endorsed the purpose of the videos and considered PLVs a useful tool to capture genuine behaviours and enable support workers to get to know the individual and not just their disability.

##### 3.2.2.3 Theme 3: Challenges Experienced During PLV Development

The most notable challenge sector professionals faced was the limited time available to produce the videos. This was due to the 4‐week time restriction imposed by the funding arrangements, alongside managing their own and the person with disability's other commitments. Time limitations were especially problematic for the storyboarding, filming and editing steps. Additional difficulties arose when working with people with severe cognitive and communication impairments. For example, goal setting and scripting were affected when participants forgot or misunderstood the purpose of the video. Sector professionals found this challenging because they wanted to ensure the person's voice was heard and everything was captured. Filming also required more planning, but professionals used strategies such as prompts or questions, which were later edited out. Finally, editing posed challenges due to unfamiliarity and the need to capture the most important messages.

##### 3.2.2.4 Theme 4: What Participants Plan to Use PLVs For

Sector professionals reported that the PLVs would be used as induction and training material for new support workers and allied health professionals (e.g., physiotherapists and occupational therapists). For some, the training focused on how the person with disability communicates or what they want and how this is possible (e.g., being included in activities). Professionals also reported different settings where people planned to use the video, such as paid employment and day programmes. Others planned to use the video as a get‐to‐know‐you resource when interviewing or meeting new workers, seeing PLVs as a tool to reduce the burden on people with disability to retell their story to new support workers or existing support workers who did not listen to them. PLVs were consistently seen as living documents that are updated when the person develops new skills, moves home or their needs and preferences change.

## 4 Discussion

The success of the pilot evaluation [1] provided cause for the extension of the implementation of the PLV intervention and training sector professionals in the use of PLV. The post‐training evaluation affirmed the value of the workshop in upskilling sector professionals to effectively collaborate with people with disability to develop a PLV, suggesting a 1‐day workshop is sufficient to teach the intervention to non‐experts. Following training, sector professionals collaborated with people with disability, including people with cognitive and communication impairments, to produce PLVs. Post‐production evaluation findings further endorsed the satisfaction, enjoyment and usefulness of the intervention, supporting the scalability potential. These results provide confidence that the training and the intervention are transferable to practice in the community to help people with disability direct their supports.

Crucially, this study suggests that PLVs support the process of getting to know the person with disability and what's important to them, which is key to the provision of quality tailored support [6, 8, 13]. Consistent with the pilot study [1], sector professionals viewed the PLV process as a useful method for enabling people with disability to set their own goals in relation to the support they receive. This finding holds important therapeutic potential for people with disability in Australia, as goal setting is critical to secure NDIS funding [2, 22, 44]. Importantly, this study reinforced that people with complex communication impairments can effectively engage with the PLV process, and alongside the pilot [1], this study shows it is feasible across disability types, including acquired neurological disability, intellectual developmental disability and autism. Two sector professionals developed PLVs with people with complex communication needs, and although it was resource‐intensive and required more planning, it was achievable with input from support workers and family members. This finding highlights another potential benefit of PLVs in improving support quality by valuing support workers' knowledge and recognising their important role, which prior research suggests enhances support quality [8, 45].

PLVs impart readily applied practical knowledge about how to support the person with disability effectively in the context of their expressed needs. Beyond the sector professionals' anticipated benefits, participant‐led resources are likely to improve sector efficiency by reducing ineffective support that fails to foster autonomy and independence [5, 6, 28]. Additionally, sector professionals suggested PLVs could aid recruitment by giving support workers a clear insight into the individual and their role in supporting them. Having better insight at the outset of employment could help increase job satisfaction and reduce the high turnover in the support workforce [42]. Positive changes in staff retention are likely to reduce the burden on family members caused by overseeing and training support workers [22, 45]. Moreover, sector professionals highlighted that PLVs could strengthen support worker accountability, the absence of which was identified as a key issue contributing to the devastating findings of the Disability Royal Commission in Australia, and is recognised as a determinant of the quality of support [3, 8, 22, 46, 47].

To improve the intervention, it is important to consider the challenges faced or anticipated by sector professionals in developing PLVs. One anticipated barrier was obtaining permission from people with disability and their family members, mainly because of the time commitment required to produce a video. A potential solution to this could be to produce resources for people with disability and their families that explain the potential benefits of PLVs for improving support quality, based on this evaluation and the pilot [1]. Additionally, it is important to establish processes to confirm ongoing permission, particularly as participants' situations change over time, necessitating continuous communication and reassessment [48]. Time constraints emerged as significant hurdles, especially during storyboarding, filming and editing, underscoring the importance of adequate planning and funding allocation for future videos. Moreover, sector professionals working with individuals with complex cognitive and communication needs faced unique difficulties supporting their active participation in decision‐making during goal setting and scripting. These challenges underscore the necessity for sector professionals to deeply understand the people they support to ensure their voices are effectively captured and represented in PLVs. This finding also reinforces the potential value of PLVs to capture the voice of people with disability, fostering a deeper understanding of the individual.

### 4.1 Practice and Policy Implications

Within Australia, the NDIS currently allocates over $7.5 billion per annum to capacity building for NDIS participants [49]. However, there is limited evidence that the interventions provided are effective at increasing the skills, knowledge and abilities of NDIS participants [33]. The NDIS Review recommended that participants with cognitive disability or complex communication support needs be connected with capacity building support and other lifelong opportunities to build decision‐making skills and experience (Action 5.2) [33]. Emerging evidence about PLVs indicates that they have the potential to provide a practical intervention for effective capacity building at scale across Australia.

PLVs aim to reduce ineffective support and foster autonomy and independence and may therefore contribute to more effective use of system resources. The findings of the PLV project thus far suggest that PLVs can positively impact the quality of support, particularly by improving support workers' understanding of the individual. Thus, we recommend funding to support further research on the implementation of PLVs, with additional resources for those with more complex needs. The finding that sector professionals need a deep understanding to address challenges faced by individuals with complex cognitive and communicationneeds further endorses the need for continuity of care both from allied health professionals and support workers [6, 8, 22, 50]. However, when continuity is not possible, PLVs can act as a bridge for sector professionals and support workers to get to know the person with disability. The pilot study, combined with the current study, evidences the humanising effect of PLVs in helping staff to see the person with disability as a person, and not merely a disability type. These findings endorse the potential for PLVs to help reduce the abuse and neglect of people with disability, as evidenced in the recent Disability Royal Commission in Australia [3, 47].

### 4.2 Future Research

The success of this study, in addition to the pilot evaluation [1], warrants extending the rollout of the PLV intervention beyond the development team to train sector professionals more broadly to collaborate with people with disability to develop PLVs. Consequently, we recommend a randomised control trial to evaluate the effectiveness of PLVs in improving the quality of support for adults with disability. Within such a trial, it would also be valuable to explore whether a streamlined version of the six‐step PLV process could maintain effectiveness, assessing the potential trade‐offs and benefits of a simplified approach. Alongside this recommendation is the need for a reliable way to measure the quality of support to effectively evaluate the impact of PLVs. Currently, no validated measure of quality support exists. A co‐designed measure of quality support, developed with people with lived experience, is needed to help evaluate the impact of PLVs and other initiatives to improve the quality of support. It would also be valuable to explore the impact of PLVs on support quality from the perspectives of people with disability and their close others and to capture support workers' experiences of viewing a PLV as part of their training. Moreover, within a large‐scale trial, further evaluation of the online resources for independent use by sector professionals, people with disability and their families should be investigated. If effective, this trial would strengthen the case for scaling PLVs across Australia by reducing the need for the 1‐day training workshop. It is also important to co‐design additional resources that guide people with disability, family members and sector professionals in using various devices and editing software to address challenges in the editing phase and improve accessibility.

### 4.3 Limitations

It is important to acknowledge the study limitations. This study only sampled a small number of sector professionals in one location in Australia. Research participants were opportunistically sampled from the sector professionals who attended the training workshop. The sample was not representative of all sector professionals who may potentially work with people to make PLVs (e.g., speech therapists). Thus, the randomised control trial should purposively seek participants from varied sector professional roles. Moreover, this study only explored the experience of producing the video and not the ongoing experience of using the video or the impact of the video on the quality of support. In addition, no pre‐workshop data collection took place. Thus, a longitudinal study is recommended with pre‐ and post‐data collection, and more open‐ended questioning to gain a deeper understanding of the feasibility of this intervention, including how people use the videos in practice, and evaluation of the impact of the videos on support quality. While people with disability were involved in the development of the PLV intervention and pilot evaluation [1], a limitation of this study is the omission to include people with disability in the training workshop. Finally, due to the time constraints, we were unable to provide participants with the opportunity to review the identified themes, which may have limited participant validation of the findings.

### 4.4 Conclusion

This study, together with the evaluation of the PLV development project [1], provides empirical evidence supporting further investment in developing and evaluating PLVs as a tool for people with disability to direct and train their support workers. Specifically, this study supports the use of a 1‐day training workshop to upskill sector professionals to collaborate with people to develop a PLV, providing a scalable process to implement the intervention. Moreover, the successful development of the videos following the training workshop provides support for the feasibility of the intervention, as well as the usability of the PLV resources. Further research is required to evaluate the effectiveness of the PLVs in improving the quality of support. However, considering the current study alongside the pilot evaluation [1], it is evident that PLVs have great potential to enhance the quality of support, are valued by people with disability and supporters, and can be feasibly developed by sector professionals when funding is allocated.

## Author Contributions

**Megan Topping:** data curation (lead), formal analysis (equal), investigation (lead), project administration (lead), writing – original draft preparation (lead), writing – review and editing (lead). **Jacinta Douglas:** conceptualisation (supporting), formal analysis (equal), funding acquisition (equal), methodology (lead), supervision (lead), writing – review and editing (equal). **Kate D'Cruz:** formal analysis (supporting), supervision (supporting), writing – review and editing (equal). **Di Winkler:** conceptualisation (lead), funding acquisition (equal), methodology (supporting), writing – review and editing (supporting).

## Conflicts of Interest

The authors declare no conflicts of interest.

## Supporting information


**Appendix 1:** Post‐workshop online survey. **Appendix 2:** 1 month post workshop interview schedule. **Appendix 3:** Post‐production interview schedule.

## Data Availability

The authors have nothing to report.
