# Supplementary material for: Evaluating the Feasibility of the Participant‐Led Video Intervention to Train Support Workers From the Perspective of Disability Sector Professionals
Source: Health Expect. 2025 Sep 17;28(5):e70432. doi: 10.1111/hex.70432 (PMC12441804; doi:10.1111/hex.70432)
Supplement: Supplementary file 1 — Appendix 1: Post‐workshop online survey. Appendix 2: 1 month post workshop interview schedule. Appendix 3: Post‐production interview schedule. [file HEX-28-e70432-s001.docx]

**Appendix 1: Post-workshop online survey**

1. What is your role or occupation?
2. How did you hear about this training?
3. Please rate your understanding of how to make a Participant Led Video before the training
4. Please rate your understanding of how to make a Participant Led Video after the training
5. Please rate your confidence in using the Participant Led Video resources in future
6. Please rate your confidence in supporting a person to make their own Participant Led Video in future
7. What information did you find most useful at today’s training? Why?
8. What information did you find least useful at today’s training? Why?
9. Did you hear anything that surprised you today? Please elaborate.
10. Was there enough opportunities to ask questions?
11. What would you like to spend more time on in future workshops?
12. What actions are you going to take after completing this workshop?
13. How likely are you to make a Participant Led Video in the next three months?
14. Who are you going to invite to make a Participant Led Video?
15. To what extent would you recommend this training on Participant Led Videos to other section professionals?
16. Can the Summer Foundation research team interview briefly about today’s training workshop via telephone? If yes, please type initials
17. Would you like to provide any additional feedback to assist us to improve our training or the resources provided?

**Appendix 2: 1 month post workshop interview schedule**

1. How would you rate your satisfaction with the training workshop?

| ***Satisfaction*** | ***1***  ***Very Low*** | ***2***  ***Low*** | ***3***  ***Medium*** | ***4***  ***High*** | ***5***  ***Very High*** |
| --- | --- | --- | --- | --- | --- |

1. How would you rate the usefulness of the training workshop?

| ***Usefulness*** | ***1***  ***Very Low*** | ***2***  ***Low*** | ***3***  ***Medium*** | ***4***  ***High*** | ***5***  ***Very High*** |
| --- | --- | --- | --- | --- | --- |

1. How would you rate your overall enjoyment of the training workshop?

| ***Enjoyment*** | ***1***  ***Very Low*** | ***2***  ***Low*** | ***3***  ***Medium*** | ***4***  ***High*** | ***5***  ***Very High*** |
| --- | --- | --- | --- | --- | --- |

1. How would you rate your confidence following the workshop to support someone with cognitive and/or communication impairments to produce a Participant Led Video?

| ***Confidence*** | ***1***  ***Very Low*** | ***2***  ***Low*** | ***3***  ***Medium*** | ***4***  ***High*** | ***5***  ***Very High*** |
| --- | --- | --- | --- | --- | --- |

1. How would you rate the overall quality of the training course?

| ***Overall quality*** | ***1***  ***Very Low*** | ***2***  ***Low*** | ***3***  ***Medium*** | ***4***  ***High*** | ***5***  ***Very High*** |
| --- | --- | --- | --- | --- | --- |

1. What were the highlights of the training course?
2. What do you think could be done to improve the training?
3. Will you be able to implement the training and support someone to produce a video over the next year?
4. If yes, how many people do you expect to be able to support over the next year?
5. What barriers do you expect to face in implementing the training?
6. Is there anything else you would like to share about the training workshop or Participant-Led Videos generally?

**Appendix 3: Post-production interview schedule**

Introduction

1. Thank participant for taking part in the project and this interview
2. Introduce self & review purpose of project

It’s important that people with disability have choice and control when it comes to choosing and training their support workers. The aim of the project is to evaluate the process and experience of creating the videos from the perspective of the sector professionals trained in the Participant Led Video process.

1. Explain interview and questionnaire process

You collaborated with _____________ to make a video describing and showing support workers how to support him/her in his/her life. We wanted to give you the opportunity of letting us know how it was from your perspective.

Making the video: Your involvement

- Can you just give me some background about how you were involved in the video production process?

Steps along the way

- There was **goal-setting and video-planning**. This step involved talking about what makes a good day for ____________ and what a support worker can do so ______________ has a good day each day. During this step ____________chose what he/she wanted to talk about in the video.

Can you tell me about what ______________chose to talk about in the video (and why you think ______________ chose that particular thing?).

How would you rate your level of satisfaction with this step in the process? (1 very low, 2 low, 3 medium, 4 high, 5 very high)

How would you rate your level of enjoyment with this step in the process? (1 very low, 2 low, 3 medium, 4 high, 5 very high)

- Then the next step was what we called **scripting and storyboarding the video**. In this step ______________ worked with you to plan how to say what he/she needed to when the video was being filmed.

Can you tell me how this step was from your perspective?

How would you rate your level of satisfaction with this step in the process? (1 very low, 2 low, 3 medium, 4 high, 5 very high)

How would you rate your level of enjoyment with this step in the process? (1 very low, 2 low, 3 medium, 4 high, 5 very high)

- In the next step, ______________ completed **filming the video**.

Can you tell me how this step was from your perspective?

How would you rate your level of satisfaction with this step in the process? (1 very low, 2 low, 3 medium, 4 high, 5 very high)

How would you rate your level of enjoyment with this step in the process? (1 very low, 2 low, 3 medium, 4 high, 5 very high)

- During the next step ______________ **watched his/her video and chose which parts were ok to share publicly**.

Can you tell me how this step was from your perspective?

How would you rate your level of satisfaction with this step in the process? (1 very low, 2 low, 3 medium, 4 high, 5 very high)

How would you rate your level of enjoyment with this step in the process? (1 very low, 2 low, 3 medium, 4 high, 5 very high)

- Now at the end of the process, ______________ has a video he/she can use with support workers.

Can you tell me what you think about the video?

Has ______________ shown it to support workers? (which ones?)

Do you know what they thought about the video?

How would you rate your level of satisfaction with the video? (1 very low, 2 low, 3 medium, 4 high, 5 very high)

How would you rate the usefulness of the video? (1 very low, 2 low, 3 medium, 4 high, 5 very high)

Now having been part of this process with ______________, how likely are you to recommend this approach for other people like _____________? (1 not at all - 10 very likely)
